# Human Papillomavirus Detection in Head and Neck Squamous Cell Carcinomas at a Tertiary Hospital in Sub-Saharan Africa

**DOI:** 10.1155/2019/2561530

**Published:** 2019-04-02

**Authors:** Evans Aboagye, Francis Agyemang-Yeboah, Babatunde Moses Duduyemi, Christian Obirikorang

**Affiliations:** ^1^Department of Molecular Medicine, Kwame Nkrumah University of Science and Technology, Kumasi, Ghana; ^2^Spectra Health Imaging and Interventional Radiology, Kumasi, Ghana; ^3^Department of Pathology, Kwame Nkrumah University of Science and Technology, Kumasi, Ghana

## Abstract

Fewer studies have been done over the years to establish the association of human papillomavirus (HPV) with head and neck squamous cell carcinoma (HNSSC) within the subregions of sub-Saharan Africa, and thus this study was designed to investigate the presence of HPV in HNSCC at a tertiary hospital in Ghana, providing additional evidence on the need to explore similar studies in other subregions. A retrospective cross-sectional study was employed to investigate the presence of the DNA of HPV genotypes in HNSCC archived tissue. A total of 100 HNSCC cases were classified as suitable for HPV genotyping. HPV-DNA was detected in 18% of the HNSCC cases, with 17 being HPV-16 and 1 dual infection with HPV-16 and HPV-18. HPV was prevalent in 50% of oropharyngeal cancers, 27% of laryngeal cancers, and 23% of oral cavity cancers. HPV E6/E7 oncogenic DNA was found in 18% of the HNSCC cases, with HPV-16 being the predominant genotype present. The pattern of HPV association was similar to earlier reported studies, recording a higher prevalence in oropharyngeal cancers, followed by laryngeal cancers and oral cavity cancers.

## 1. Introduction

Head and neck cancers comprise a broad range of tumors that have been grouped as an entity, based on common etiology, anatomy, and sensitivity to treatment [1]. These malignancies arise from the upper aerodigestive tract and include tumors of the oral cavity, nasal cavity, paranasal sinuses, pharynx, and larynx [2]. About 95% of head and neck cancer (HNC) originate from squamous cells and hence are usually referred to as head and neck squamous cell carcinoma (HNSCC) [3]. Alcohol and tobacco are considered as the leading predisposing factors for developing this type of tumor, but now Human Papillomavirus (HPV), principally HPV-16, has also been identified to be associated with a subset of HNC, particularly oropharyngeal cancers (OPCs) [4, 5], with recent studies reporting a rise in HPV-induced HNC and a decline or stability in tobacco- and alcohol-induced HNC [6].

According to the Global Cancer Statistics 2018 (GLOBOCAN 2018) [7], the number of new cases of cancers of the oral cavity, larynx, and oropharynx in Africa was 13 613, 10 058, and 2 514, respectively. Furthermore, the numbers of these cancers in Ghana were 244, 188, and 93 for cancers of the oral cavity, larynx, and oropharynx, respectively. Moreover, statistics from the worldwide cancer registry data have shown an increasing incidence of HPV-positive in oral and oropharyngeal cancers, reporting a higher incidence among young men (<60 years) [8]. A recent study by Casellsagué et al. [9], which included 1374 pharyngeal cancers, 1264 oral cavity cancers, and 1042 laryngeal cancers from 29 countries, reported the presence of HPV-DNA in 22.4% of oropharyngeal cancers, 4.4% in oral cavity cancers, and 3.5% in laryngeal cancers. This shows that even small incidence of HPV-positivity in non-OPC HNC cases can translate to a high number of cases since the incidence rates of oral cavity cancers and laryngeal cancers are high. In addition, a recent meta-analysis of 148 studies involving 12,163 HNSCC cases showed the existence of HPV-DNA in 31.5% of tumors with greater prevalence in the oropharyngeal squamous cell carcinomas (SCCs) (45.8%), followed by oral squamous cell carcinomas (24.2%) and laryngeal squamous cell carcinomas (22.1%). The most prevalent HPV genotype was HPV 16, which accounted for over 82% of all HPV-positive cases [10, 11]. Fewer studies have been reported in sub-Saharan Africa, but a recent study recorded the presence of HPV-positive DNA in 15 (19.23%) out of 79 cases of HNSCC [12].

HPV transmission in head and neck cancer has been linked primarily to oral sexual behaviors [13–15]. Since sexual behaviors are correlated, other measures of sexual behavior, such sexual debut at earlier stages of life, genital warts history, and multiple sex partners, have been linked with HPV-positive HNC [14–16]. This is reinforced by recent studies in the US which showed that the incidence of oral sex increased from 50% in 1970 to 90% in 2006 and that majority of world population are recording their first sexual encounter at earlier stages of life and are having multiple sexual partners [17–19].

The implication of HPV in HNCs has led to the creation of a new subject profile in the hospital, as noted in a study by Goon et al., [20] that subjects now present with a weak or no history of alcohol or tobacco and at a much younger age. A six-year review of HNC at a tertiary hospital in Ghana reported a similar clinical profile, with 167 (61.5%) out of 252 cases of HNC found in patients aged between 10 and 59 [21]. Based on this observation, this study was designed to determine the percentage of the HNSCC cases that were associated with HPV at the same study site.

## 2. Material and Methods

### 2.1. Design

A retrospective cross-sectional study was employed to investigate the existence of the DNA of HPV genotypes in head and neck squamous cell carcinoma (HNSCC) archived tissue samples retrieved from the Department of Pathology at our study site, from January 2007 to December 2016.

### 2.2. Participants

Approval for the study was obtained from the Committee on Human Research, Publications and Ethics of our study site (*CHRPE/AP/438/17)*. The study was conducted within the period of October 2016 to July 2018.

Patients who were aged 10 years and above and histopathologically diagnosed with squamous cell carcinoma of the head and neck within the specified duration (January 2007 to December 2016) were included in the study. Specimens were essentially excluded from the study for any of the following reasons: (i) no consensus in diagnosis; (ii) unavailability of tissue block; (iii) insufficient DNA for analysis as determined using Nanodrop spectrophotometer (Nanodrop Technologies, Wilmington, DE); and (iv) negative beta-globin amplification by PCR technique.

### 2.3. Data Collection

Data regarding age, sex, site, and histological diagnosis on HNSCCs were extracted from the surgical day book of the Department of Pathology.

### 2.4. Histopathological Evaluation

In consecutive order (from 2007-2016), archived, formalin-fixed paraffin embedded (FFPE) HNSCC tissue block and slides of selected cases were retrieved from the archives of the Pathology Department. The hematoxylin and eosin (H&E)-stained slides of the selected cases were then reviewed independently by two pathologists to confirm the diagnosis. With the aid of a microtome, 10*μ*m section of each selected tissue block was taken twice. For each tissue block, the microtome was thoroughly wiped and a fresh microtome blade was used. In case of suboptimal information, the additional section of a block was stained with H&E and assessed again by the pathologists. The tumors were then graded according to recommended guidelines of the American Joint Commission on Cancer [22].

### 2.5. Genomic DNA Extraction and Quality Control

Genomic DNA was prepared from a 10*μ*m FFPE tissue section using the Quick-DNA™ FFPE Kit (catalog no. D3067; Zymo Research Corp.) according to the manufacturer's instructions. There was deparaffinization of the FFPE tissue using a deparaffinization solution. This was followed by digestion using proteinase K and RNase A, and finally purification of the tissue using genomic lysis buffer, isopropanol and genomic DNA wash. The extracted DNA was quantified using a Nanodrop spectrophotometer (Nanodrop Technologies, Wilmington, DE) and stored at -20°C for future use. The DNA lysate prepared was quality controlled using human beta-globulin DNA polymerase chain reaction (PCR) as described by de Roda Husman et al., [23]. For a PCR volume of 25*μ*l, 4*μ*l of the DNA lysate and 25pmol of each human beta-globulin consensus primers PCO_3_^+^ and PCO_4_^+^ (Integrated DNA Technologies, Inc., USA) were used.

### 2.6. DNA Amplification and Genotyping

The HPV-DNA amplification and detection were performed using nested multiplex PCR as described by Sotlar et al. [24]. General primers consisting of a single consensus forward primer (GP-E6-3F) and two consensus back primers (GP-E7-5B and GP-E7-6B) (Eurogenetics, Belgium) were used to generate a PCR product of 630 base pair (bp) in the E6/E7 gene region of the HPV genome in the first multiplex PCR. DNA extracts from the first round PCR was subsequently used as template for the second multiplex PCR, which involved identification of specific HPV genotypes. Primers for identification of both high-risk HPV genotypes (HPV-16, HPV-18, HPV-31, HPV-33, HPV-35, HPV-45, HPV-51, HPV-52, HPV-56, HPV-58, and HPV-59) and low-risk HPV genotypes (HPV-6/11, HPV-42, HPV-43, and HPV-44) (Eurogenetics, Belgium) were used in 3 cocktails each containing five different primer pairs. All PCR runs had a negative control (nuclease-free water) and a positive control (HPV-16 plasmid DNA).

**Figure 1:**
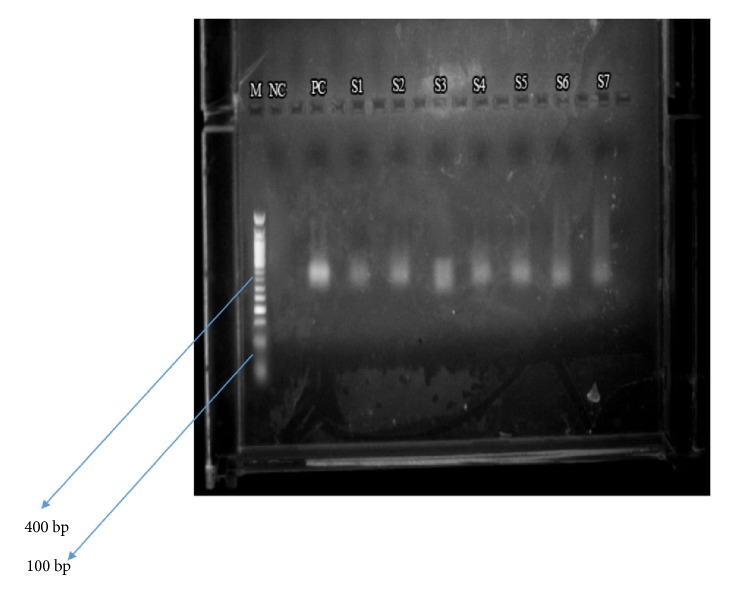
An electropherogram of 2nd nested PCR products. The 457 bp and 322 bp fragments correspond to amplified HPV-16 and HPV-18 DNA, respectively. Lane M: 50 bp molecular size marker; NC: negative control (nuclease-free water); PC: positive control (HPV-16 plasmid DNA); S1, S2, S4, S5, S6, and S7: HPV-16 DNA positive samples; S3: HPV-16 and HPV-18 DNA coinfection (a PCR product ranging from 322 bp to 457 bp).

Gel electrophoresis was employed in the analysis of the amplified products, using 2% agarose gel and 0.5*μ*g/ml ethidium bromide. Ten microlitres of each sample were added to 2*μ*l of either blue or purple (6X) gel loading dye for the electrophoresis. Fifty bp DNA molecular weight marker (New England Biolabs® Inc.) was run alongside the PCR products. The gel was prepared and electrophoresed in 1X TAE buffer using a mini gel system at 100 volts for one hour and the gel photographed over UV transilluminator [24] to generate an electropherogram (Figure 1).

## 3. Results

Within the period of January 2007 to December 2016, a total of 100 head and neck squamous cell carcinoma (HNSCC) cases were classified as suitable for HPV testing and genotyping. The mean age of the patients was 52.36 years (median age, 56 years). Majority of the patients were >56 years (51%). The distribution is shown in Table 1.

Majority of the samples were from the larynx, followed by oral cavity with hypopharynx being the least represented. Eighteen (18) out of the 100 HNSCC samples were positive for HPV-DNA. Out of the 18, 15 (83.3%) were males while 3 (16.7%) were females. The males were thus more likely to have HPV-positive HNSCC compared to the females (OR=2.45; 95% CI: 0.654-9.211). The patients were again stratified into young (<56 years) and old (≥56 years) based upon the median age of the study group. The results showed that there was a trend towards patients ≥56 years being more likely to have HPV-positive tumors (OR=1.378; 95% CI: 0.494-3.844), but this difference was not statistically significant. The distribution of the 18 HPV-positive cases according to location showed that HPV was prevalent in 50% (6 out 12) of oropharyngeal cancers, 27% (7 out of 26) of laryngeal cancers, and 22% (5 out of 23) of oral cavity cancers. Majority of the HPV-positive tumors were found in moderately differentiated samples (n=9, 50%), followed by well differentiated samples (n=5, 27.8%). The distribution is shown in Table 1.

The most common genotype found was HPV-16, present as a single infection in 17 out of the 18 cases, with one case of HPV-16 and HPV-18 coinfection, found in laryngeal cancer. None of the other 13 HPV genotypes tested for in the study was observed (6/11, 31, 33, 35, 42, 43, 44, 45, 51, 52, 56, 58 and 59). The distribution is shown in Table 2.

## 4. Discussion

The study employed a nested multiplex PCR (NMPCR) technique as described by Sotlar et al. [24] for HPV genotyping in the 100 HNSCC tissue samples. The study recorded a prevalence of 18% HPV-positive DNA tumors after analysis of the amplification products on 2% agarose gel. A similar finding has been reported in Ghana recording a prevalence of 19.2% [12]. Furthermore, Asante et al. [25] in their study in Ghana, which involved only nasopharyngeal carcinomas, also recorded HPV prevalence of 19.4%. A study by Ndiaye et al. [26] in Senegal reported a low prevalence (2.9%) of HPV in 140 HNSCC cases. Gillison et al. [4] in their study in the US on the etiological role for HPVs in HNSCC detected HPV-DNA in 62 (25%) of the 253 cases. Furthermore, Kreimer et al. [27] in a systematic review of 60 studies on HPV types in HNSCC reported an overall HPV prevalence of 25.9%. Ndiaye et al. [11] in a recent meta-analysis of 148 studies involving 12, 163 HNSCC cases also reported the prevalence of HPV-DNA in 31.5% of the tumors. The study further revealed that male subjects were at a higher risk of HPV infection compared to their female counterparts (OR=2.45), even though the prevalence estimates were not statistically significant. Similar findings have been recorded by other studies [12, 14, 28]. Furthermore, this study found a trend towards older patients ≥56 years having a higher chance of harboring HPV associated HNSCCs than younger patients (OR=1.378). However, there was no statistical significance between the oncogenic viral status with either gender or age in the selected HNSCC cases (Table 1).

HR-HPV type 16 and type 18 were the only HPV genotypes detected in this study, although HPV-6, HPV-11, and HPV-33 have been reported in other studies of HNSCCs albeit rarely [29, 30]. Moreover, the study affirms the dominance of HPV-16 and the relative rarity of HPV-18 in HPV-positive HNSCCs (compared to HPV-positive cervical carcinomas) as recorded in other works [9, 11, 28, 29]. The study also recorded a higher association of HPV with oropharyngeal carcinomas, followed by laryngeal carcinomas. Earlier studies done in HPV in HNSCC have also recorded a higher association of HPV with oropharyngeal carcinomas compared to other sites [9, 14, 28]. Also, majority of the HPV-positive samples were high-grade tumors (grade II and III). This was in line with the observation by Gillison et al. [4] but contrary to that of Kaba et al. [12] who recorded a higher percentage of their HPV-positive samples in low-grade tumors (grade I). The study however found no association of tumor grade with HPV infection, as reported in other studies, although poorly differentiated tumors (grade III) have been postulated to be 2.4 times more likely to harbor HPV infection [4, 31]. It must however be noted that the presence of HPV-DNA in HNCs is not enough prove of viral causation, as it might just reflect a transient infection unrelated to the carcinogenic process [11, 32]. Thus, it is necessary to explore other biomarkers such as HPV E6/E7 mRNA or p16^INK4a^ which have been associated with HPV-induced carcinogenesis. Since the current study did not explore any biomarker associated with HPV, we could not conclude with certainty that the HNC samples that harbored HPV-DNA were actually caused by the HPV virus.

The clinical and prognostic implication of HPV-positive HNSCCs have been established, and there is a general consensus among the scientific community that it is distinct from those of conventional, alcohol- and tobacco-related HNSCCs [33]. Majority of the studies done so far on HPV in HNSCC have shown that HPV-positive HNSCC patients, particularly OPC patients, have a better response to therapies and have a higher survival rate compared to their HPV-negative counterparts [33, 34]. Thus, clinical trials on HNSCC management in recent years have focused on the possibility of reducing the amount of therapy required by HPV-positive patients, with the potential of reducing long-term side effects in a younger patient population.

## 5. Conclusions

The study has shown a prevalence of HPV-positive DNA rate of 18% in 100 cases of HNSCC, revealing a preponderance in males. The study also affirmed the dominance of HPV-16 and the relative rarity of HPV-18 in HPV-positive HNSCC as reported by other studies. The observed pattern of HPV association in our study population was similar to earlier reported studies, recording a higher prevalence in oropharyngeal cancers, followed by laryngeal cancers and oral cavity cancers. However, this was a small study and therefore requires a large scale population studies in sub-Saharan Africa to confirm our findings.

## Figures and Tables

**Table 1:** Characteristics of the study population and HPV status.

Characteristics	Study Population	HPV- (+)	HPV- (-)	Fisher's Exact Test
	(n= 100)	(n=18)	(n=82)	P value
*Gender*				
Male	70	15	55	0.608
Female	30	3	27	
*Age*				
<56 years	51	8	43	0.162
≥ 56 years	49	10	39	
*Tumour site*				
Oral cavity	23	5	18	
Oropharynx	12	6	6	
Hypopharynx	1	0	1	
Larynx	26	7	19	0.300
Nasopharynx	5	0	5	
Nasal cavity and paranasal sinuses	18	0	18	
Salivary gland	4	0	4	
Undetermined^*∗*^	11	0	11	
*Tumour Grade*				
Well differentiated	21	5	16	
Moderately differentiated	43	9	34	0.302
Poorly differentiated	36	4	32	

Undetermined*∗*- written as head and neck without specification.

**Table 2:** Human papillomavirus genotypes (n=18).

HPV Genotype	No. (%)
HPV-16	17 (94.4)
HPV-16, HPV-18	1 (5.6%)

## Data Availability

Data is available upon request but subject to ethical approval of the Committee on Human Research, Publications and Ethics at our study site.
